# Violence against Canadian Women

**DOI:** 10.1186/1472-6874-4-S1-S22

**Published:** 2004-08-25

**Authors:** Marsha M Cohen, Heather Maclean

**Affiliations:** 1 Centre for Research in Women's Health, 790 Bay St., 7th Floor, Toronto, Canada; 2 Centre for Research in Women's Health, 790 Bay St., 7th Floor, Toronto, Canada

## Abstract

**Health Issue:**

Exposure to violence as children or as adults places a woman at higher risk of poor health outcomes, both physical and psychological. Abused women use more health care services and have poorer social functioning than non-abused women. Knowledge of the prevalence of violence against women, and of which women are at risk, should assist in the planning of services for abuse prevention and treatment of the health consequences of abuse.

**Key Findings:**

The highest rates of any partner violence were in Alberta (25.5%) and British Columbia (23%). The lowest rates were in Ontario (18.8%). Women aged 15–24 had the highest rates in all regions in Canada, compared with older women. Aboriginal women in Manitoba/Saskatchewan and Alberta had higher rates of violence (57.2% and 56.6% respectively) than non-Aboriginal women (20.6%). Lower rates of partner-related violence were reported among women not born in Canada (18.4%) than among Canadian-born women (21.7%). Visible minority women reported lower rates of lifetime sexual assault (5.7%) than non-visible minority women (12.3%). Perceptions of violence may vary by ethnicity.

**Data Gaps and Recommendations:**

More information is required concerning the prevalence of violence among Aboriginal women, immigrant and refugee women, women with disabilities, lesbian women and pregnant women. Future national population-based surveys need better questions on the health consequences of violence and related resource utilization. Further research is needed to identify the health care system's role in prevention, management and rehabilitation as they relate to violence against women. Future programs and policies must be based on valid, reliable and comprehensive empirical data.

## Background

The definitions of violence and health used in this chapter are those from the United Nations and the World Health Organization (WHO). The United Nations definition of gender-based violence includes any act "that results in, or is likely to result in, physical, sexual or psychological harm or suffering to women, including threats of such acts, coercion or arbitrary deprivations of liberty, whether occurring in public or private life." The WHO gives the definition of health as follows: "Health is a state of complete physical, mental and social well-being and not merely the absence of disease or infirmity."

## Prevalence of Violence Against Women

In the literature, rates of violence against women vary, depending on how sexual assault or intimate partner violence is defined (e.g. physical, emotional or sexual abuse), the way the questions are posed (the number of and detail in the questions), and the way in which the questions are asked (for example, written survey or face-to-face interview). Other differences may be due to the characteristics of the women studied, the use of single versus multiple interviewers, and whether women are questioned about current or past abuse.

**Figure 1:**
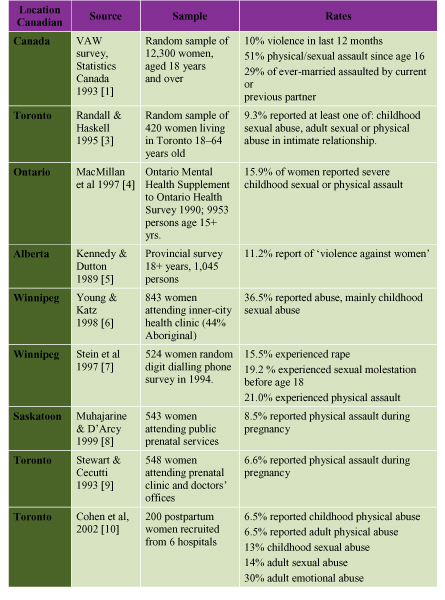
Prevalence of Violence Against Women: Canada

**Figure 2:**
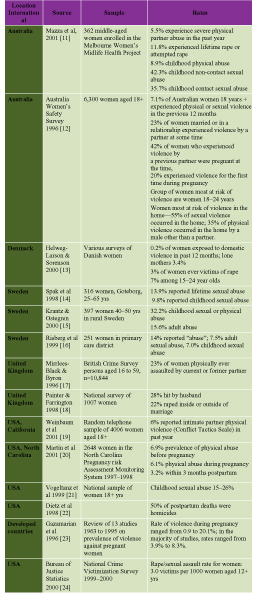
Prevalence of Violence Against Women: Developed Countries

**Figure 3:**
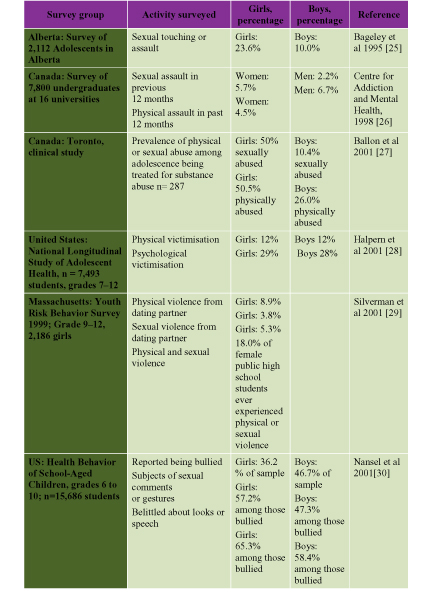
Violence and Victimization Studies among Adolescents: Canada and United States

In Canada, two national surveys of violence against women[1,2] and a small number of local or regional studies have been carried out (see Figure 1). Because of methodological and population differences it is difficult to make direct comparisons of rates of violence between Canada and other developed countries. A sample of studies from Sweden, the United Kingdom and the United States, summarized in Figure 2, shows rates of violence to women. Recent studies of violence against adolescent and younger women are summarized in Figure 3.

## Health Effects of Violence Against Women

Women subject to abuse have high rates of physical illnesses: higher rates of pelvic inflammatory disease, STDs including HIV/AIDS, bladder infections, chronic pelvic pain and other genitourinary problems[31], gynecological problems[32] and higher rates of abnormal Pap tests[6]. Abused women also have higher rates of musculoskeletal pain[33] and gastrointestinal disorders[34].

Among adolescent girls, physical and sexual violence during dating is associated with an increased risk of substance use, unhealthy weight control measures (e.g. use of laxatives, vomiting), sexual risk behaviours, pregnancy and suicide attempts[29]. Early-onset smoking behaviours are also associated with emotional, sexual or physical assault[35].

Women who had reported rape or physical assault reported severely decreased quality of life and limitations of educational and financial attainment more than a decade later[36]. Even exposure to "low-severity" violence has been found to be associated with physical and psychological health problems inwomen[37]. Injuries from abuse may be very severe and sometimes fatal. When the definition of pregnancy-related mortality is widened to include homicide, the leading cause of death during pregnancy or within one year after delivery is homicide (20% of pregnancy-related deaths)[38].

Pregnant women who were abused are more likely to use alcohol, cigarettes and drugs than non-abused women[9,39,40] and are more likely to suffer from mental disorders during pregnancy[41]. Pregnant women subjected to abuse often delay seeking antenatal care[42,43]. Abuse in pregnancy is a factor for poor obstetric outcomes, such as miscarriage, neonatal deaths[44], preterm labour[45] and low birth-weight infants[46]. Abuse is also a factor in unintended or unwanted pregnancies[47].

Several studies now confirm the relation between abuse and poor mental health, especially depression [48-50]. Significant associations have been found between childhood sexual abuse and both subsequent smoking, and alcohol and drug dependence[14,35,51]. Women who have been sexually abused are also more likely to report lifetime use of prescription psychoactive drugs and illicit drugs, and sexual dysfunction[52]. They have higher lifetime reported rates of suicide attempts and post-traumatic stress symptoms; women sexually assaulted before the age of 16 are more likely to attempt suicide[53,54].

## Gender Impact

Surveys of cohabiting couples (married and non-married) have used questions from the Conflict Tactics Scale[55]. These surveys ask respondents whether their partner has ever thrown something, pushed, grabbed or shoved, slapped, kicked, bitten or hit, beat up, choked, burned, forced sex, or threatened to use or used a gun or knife. These studies have shown that "any" violence occurs about equally between men and women, but the pattern of violence differs. Women reported that they had been subjected to more severe violence (e.g. were beaten up, had forced sex), whereas men were more likely to have been pushed or slapped[2]. Because of their generally smaller bodies, women are more likely to be injured than men in a violent confrontation. Thus it is important to consider the context of the violence.

### Effect of Violence Against Women on Health

#### Care Utilization

Women who have experienced abuse use health services at rates higher than non-abused women. These include higher rates of physician visits, emergency room visits and hospitalizations, and they report poorer ratings of their health[49,56-59].

Thus, experiencing abuse, whether as a child or an adult, places women at higher risk of poor health outcomes, both physical and psychological. These women show higher use of the health care system and poorer social functioning, consequences that have important implications for resource allocation and direction of the health care system. Estimates of the annual cost of medical treatment of abused women in Canada range from $408 million[60] to $1.5 billion[61], and estimated in-patient hospital costs related to violence range from $37.8 million to $70.7 million[1].

## Method

In this chapter data from the 1999 General Social Survey (GSS) were analyzed. The GSS is a national survey that is designed to monitor the attitudes and experiences of Canadians on a wide range of issues. The 1999 GSS covered a broad range of topics related to violence and victimization.

For the 1999 GSS, approximately 26,000 Canadians were interviewed between February 1999 and December 1999. Intimate partner violence was assessed using a version of the Conflict Tactics Scale. Respondents were women and men 15 years of age or older in the 10 provinces. Excluded were those who lived full time in institutions; those from the Yukon, Northwest Territories, and Nunavut; those who did not have a telephone (approximately 2% of the population); those not speaking French or English; and those who were homeless.

Respondents for the survey were selected using a process of Random Digit Dialling. Interviews were conducted through computer-assisted telephone interviewing. The number of women surveyed for the 1999 GSS in Canada was 14,269. Responses were weighted to represent the population of non-institutionalized women 15 years of age or older living in Canada. Fewer than 15 women in a particular cell was deemed insufficient for statistical analysis, and this cell remained blank.

### Analysis

For this study, two main categories of violence were used. The first was "violence from a current partner" or "violence from a former partner" within the previous 12 months or 5 years. Because of small numbers of respondents, we have combined responses from the previous 12 months and previous 5 years to yield larger numbers for statistical analyses. As well, we combined responses based on current partner with those from former partner. The second main category of questions dealt with "lifetime sexual assault" from a non-partner.

The types of violence included in calculation of rates were non-severe physical violence, severe physical violence, "any physical or sexual violence" (non-severe plus severe), emotional abuse, emotional or financial abuse, and "any" intimate partner abuse (physical, emotional or financial). We also examined the rate of lifetime sexual abuse.

The 1999 GSS contained several questions relating to the use of health care professionals as well as the use of drugs for anxiety, depression or sleep among those who had experienced abuse. The number who had experienced abuse was too small to carry out further analyses on these health-related variables, except the use of drugs.

Finally, we determined the rates of violence in regions of Canada by factors such as ethnicity, education, income and presence of children, and compared the rates of violence between women who had the characteristic of interest with those who did not (for example, the rate of violence among Aboriginal as compared with non-Aboriginal women), using the z-test and *p *< 0.05 as the significant value.

## Results

### Overall Rates

**Figure 4:**
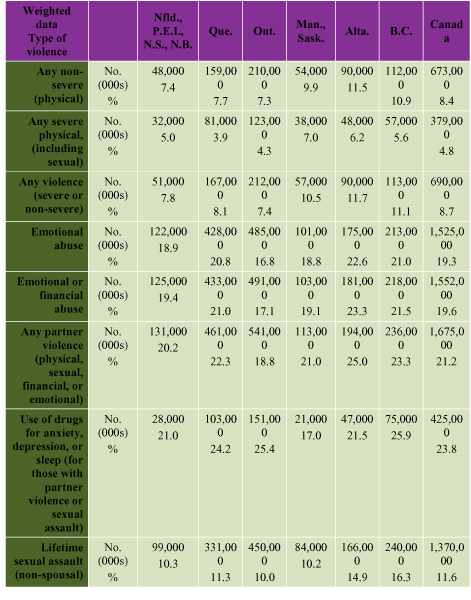
**Rate of Violence (%) Against Women by Partner or Ex-partner in the Previous 5 Years, Canada and the Regions, 1999. **Source: Statistics Canada, General Social Survey, 1999

The rates of violence reported by women respondents in the 1999 GSS are found in Figure 4; data are presented by type of violence, for regions (provincial groupings) and for all of Canada. The proportion of respondents has been converted to the estimated number of women who would have experienced the various abusive situations.

Any non-severe partner violence was reported by 8.4% of Canadian women. The highest rate was seen in Alberta (11.5%) and the lowest rate in Ontario (7.3%). The prevalence of severe violence was lower than that of non-severe violence. The reported rate for Canada was 4.8% (range 3.9% in Quebec to 7.0% in Manitoba/Saskatchewan). Combining non-severe and severe violence, the rate for Canada was 8.7%, the highest rates occurring in Alberta and the lowest in Ontario.

Reported emotional abuse (with or without financial abuse) was found to be more prevalent than physical or sexual abuse: 19.3% reported emotional abuse, and this did not vary greatly among the regions, ranging from 22.6% in to 16.8% in Ontario.

Summing up the various modes of intimate partner abuse (physical, sexual, emotional and financial), the reported rate of "any" abuse was 21.2% among Canadian women. This did not vary much across the provinces, the rates ranging from 18.8% (Ontario) to 25.0% (Alberta).

Women were also asked about their lifetime experience with non-intimate-partner sexual abuse. Overall, 11.6% stated that they had been exposed to sexual abuse. This varied from 10.0% in Ontario to 16.3% in British Columbia.

Of women who claimed at least one mode of abuse, 23.8% reported using drugs for anxiety, depression or sleeping. The use of these medications varied from a low of 17.0% in Manitoba/Saskatchewan to a high of 25.9% in British Columbia.

### Place of Birth

**Figure 5:**
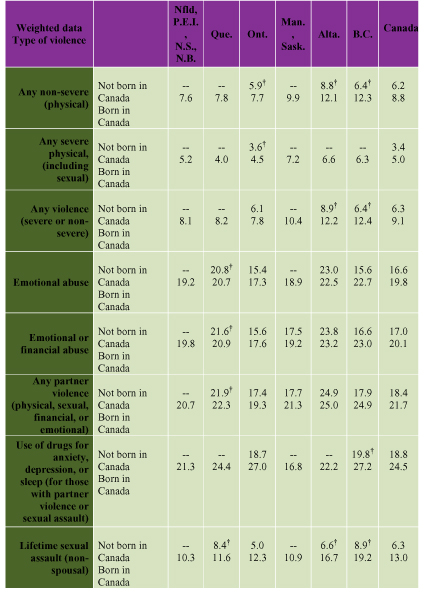
**Rate of Violence (%) Against Women by Partner or Ex-partner in the Previous 5 Years, by Place of Birth, Canada and the Regions, 1999. **Source: Statistics Canada, General Social Survey, 1999.

The data were analyzed according to whether the respondent had been born in Canada or elsewhere (Figure 5).

Women born in Canada reported higher rates of all types of violence than those not born in Canada: for example, the rate of non-severe violence was 8.8% as compared with 6.2% among those not born in Canada (*p *< 0.01). Although this pattern was seen for all regions with a sufficiently large number of respondents, only in British Columbia was the rate of non-severe violence significantly greater among women born in Canada than among those not born in Canada (12.3% versus 6.4%, *p *< 0.01). There was no statistically significant difference by place of birth (*p *> 0.05) among women who reported at least one form of abuse and used medication for anxiety, depression or sleep.

Lifetime sexual assault was also less prevalent among women who were not born in Canada for all regions for which data were available (*p *< 0.001) except Quebec, where the difference was not statistically significant (Figure 5). In Alberta, the rate of lifetime sexual abuse was 16.7% among Canadian-born women and 6.6% among women born elsewhere (*p *< 0.001). In Ontario, women born in Canada were nearly twice as likely to report lifetime sexual violence than women born elsewhere (*p *< 0.001).

### Visible Minority Status (Figure 6)

For most regions except Ontario, there were too few respondents who identified themselves as a "visible minority" for full analysis. Overall, for Canada there was little difference in the rate of non-severe physical violence reported by women who did not identify themselves as a visible minority (8.4%) compared with women who did (7.9%) (*p *> 0.05).

For Canada, Ontario and Alberta, there were no significant differences between visible minority women and non-visible minority women in the rate of emotional abuse, or in the rate of emotional or financial abuse. However, in Quebec, both forms (emotional abuse and emotional or financial abuse) were more common among visible minority respondents than non-visible minority respondents (*p *< 0.05).

Overall, no significant differences between visible and non-visible minority women were found in the use of medications. The rate of lifetime sexual assault was reported to be less among visible minority women in Canada overall (*p *< 0.001), in Ontario (*p *< 0.001) and in British Columbia (*p *< 0.001) than among non-visible minority women.

### Aboriginal Women (Figure 7)

**Figure 7:**
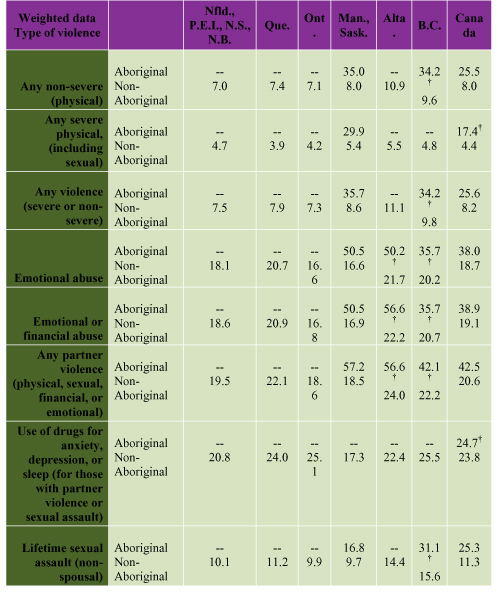
**Rate of Violence (%) Against Women by Partner or Ex-partner in the Previous 5 Years, by Self-identified Aboriginal Status, Canada and the Regions, 1999. **Source: Statistics Canada, General Social Survey, 1999

For the eastern provinces and regions, there were too few respondents in the General Social Survey who identified themselves as Aboriginal for analysis. However, the numbers in western Canada were large enough to examine some of the types of violence among these women (Figure 7).

The rates of all types of violence were higher among Aboriginal women than among women who did not identify themselves as Aboriginal. For example, the reported rates of any intimate partner violence in Aboriginal women compared with non-Aboriginal women were 57.2% and 18.5% in Manitoba/ Saskatchewan (*p *< 0.05), 56.6% and 24% in Alberta (*p *< 0.05), and 42.1% and 22.2% in British Columbia (*p *< 0.05). The use of medications for victims of violence was about the same for Aboriginal and non-Aboriginal women.

A higher proportion of Aboriginal than non-Aboriginal women reported lifetime sexual assault by a non-partner in British Columbia (31.1% versus 15.6%, *p *< 0.05). In Manitoba/Saskatchewan, the rate of sexual assault was not statistically different between Aboriginal and non-Aboriginal women.

### Urban/Rural (P.E.I.) Status (Figure 8)

In general, there were no statistically significant differences between women living in urban areas and those living in rural areas in the rates of partner violence or sexual assault. The use of medication for anxiety, depression and sleep was also very similar for urban and rural women.

### Age (Figure 9)

**Figure 9:**
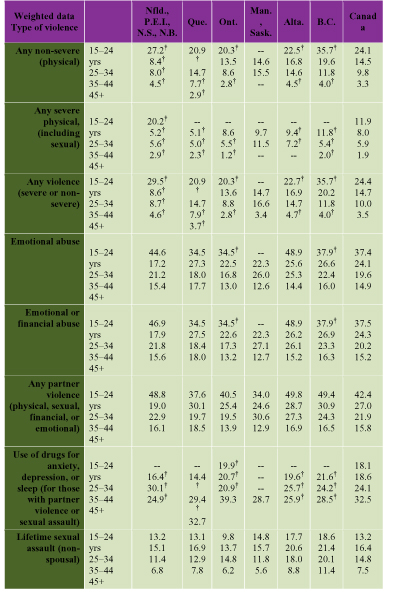
**Rate of Violence (%) Against Women by Partner or Ex-partner in the Previous 5 Years, by Age Group of Respondents, Canada and the Regions, 1999. **Source: Statistics Canada, General Social Survey, 1999

Rates of all types of intimate partner violence were related to the age of the respondent (Figure 9). Younger women (15 to 24 years) were significantly more likely to report violence than older women (over age 45 years) (*p *< 0.05). Women aged 25 to 34 years were also significantly more likely to report all types of partner violence than women 35 to 44 years of age (*p *< 0.05).

For non-severe violence an age gradient was found, in that the youngest age group of women had the highest rate (24.1% for Canada) and women over 45 years had the lowest rates (3.3%). Similar patterns were seen for all provinces and regions. Similar age gradients were seen when severe violence was considered.

The use of medications among victims of violence was lower for younger women than for older respondents: 18.1% as compared with 32.5% (*p *< 0.001).

Overall in Canada, younger women aged 15 to 24 were significantly less likely than women aged 25 to 34 years to claim a history of lifetime sexual assault (13.2% versus 16.4%, *p *< 0.05). This pattern was seen for all provinces and regions. Rates of lifetime sexual assault were significantly higher among younger women in Alberta (*p *< 0.05) and British Columbia (*p *< 0.01) than among younger women in Ontario.

### Activity Limitations (Figure 10)

**Figure 10:**
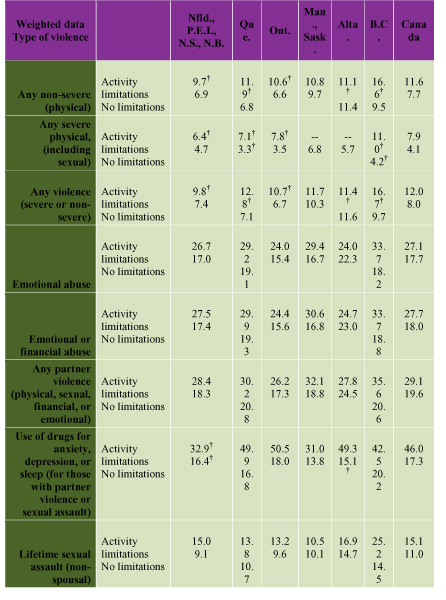
**Rate of Violence (%) Against Women by Partner or Ex-partner in the Previous 5 Years, by Self-identified Activity Limitations, Canada and the Regions, 1999. **Source: Statistics Canada, General Social Survey, 1999

No direct questions were asked about disability in the 1999 GSS, but there were questions about "activity limitations" as defined by the respondent. For this analysis, women who responded positively to the item "Does a long term physical or mental condition or health problem reduce the amount or the kind of activity that you can do at home, at school, at work or in other activities?" were compared with women who did not state an activity limitation (Figure 10).

Overall for Canada, respondents with a reported activity limitation were significantly more likely to report all types of partner violence than those without an activity limitation (*p *< 0.01). This was seen for non-severe, severe and emotional abuse. For most provinces/regions, the rate of violence was higher among women with activity limitations, especially in British Columbia. Rates of any physical or sexual partner violence were 16.7% for British Columbia women with activity limitations as compared with 9.7% for women without activity limitations (*p *< 0.05).

Women reporting activity limitations who were victims of abuse were very much more likely to use medications. In Ontario, 50.5% of women with activity limitations used medications as compared with 18.0% of women without activity limitations (*p *< 0.001).

Lifetime sexual assault was significantly more common among women reporting activity limitations in the Atlantic provinces (*p *< 0.05), Ontario (*p *< 0.05), and British Columbia (*p *< 0.01), compared with women without activity limitations. The greatest differences were seen in British Columbia: 25.2% as compared with 14.5% of women without activity limitations (*p *< 0.01).

### Parental Status (Figure 11)

**Figure 11:**
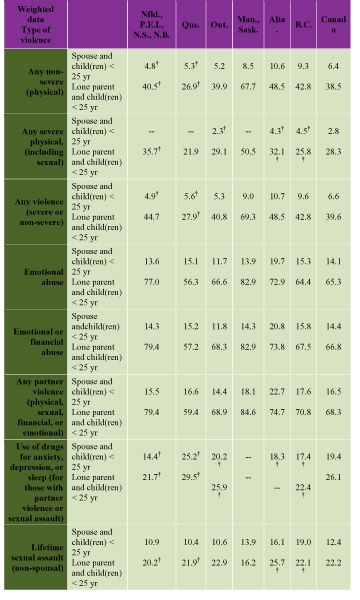
**Rate of Violence (%) Against Women by Partner or Ex-partner in the Previous 5 Years, by Parental Status, Canada and the Regions, 1999. **Source: Statistics Canada, General Social Survey, 1999

In this analysis, respondents who lived with a partner and children under 25 years of age were compared with respondents who were lone parents having children under 25 years of age in the household (Figure 11). In Canada, lone parents were significantly more likely to report an episode of partner violence than women respondents who lived with a partner and children (*p *< 0.001).

Rates of non-severe violence were very high for lone parents with children. In Manitoba/Saskatchewan, 67.7% of lone-parent respondents claimed non-severe physical violence from an intimate partner in the previous five years. This is compared with a rate of 8.5% for respondents living with a partner and children (*p *< 0.01). Rates of severe violence were also very high for lone-parent respondents. For example, in Manitoba/Saskatchewan, 50.5% of lone-parent respondents reported severe intimate partner violence in the previous five years.

Emotional abuse was also often found to be prevalent for lone parents. In Alberta, for example, 73.8% of respondents who were lone parents reported emotional or financial abuse compared with 20.8% for respondents who lived with a partner and children (*p *< 0.001).

Rates of any partner violence for lone parents ranged from 59.4% in Quebec to 84.6% in Manitoba/Saskatchewan. In Canada, victims of violence who were lone parents were equally likely to use medications as respondents living with a partner (*p *> 0.05).

In Canada, women respondents who were lone parents also had a higher rate of lifetime sexual ssault, which was about twice as high as that of women who currently lived with a partner and children (*p *< 0.001).

### Presence or Absence of Partner in the Household (Figure 12)

In this analysis, the rate of violence among respondents who did not have a partner in the household was compared with the rate among those who had a married partner in the household and the rate of those who had a common-law partner. There were too few same-sex households for analysis.

Women who were currently not living with a partner reported higher rates of intimate partner violence than women who lived with a common-law partner or were married. For example, in Quebec, the rate of non-severe physical violence among respondents was 23.7% for women with no current partner living in the household, compared with 11.4% for those with a common-law partner (*p *< 0.001) and 2.0% for those currently married (*p *< 0.001). Similar patterns were seen for all the types of physical violence examined.

Rates of any partner violence were very high among women who were unpartnered. For example, in Alberta, 63.0% of these respondents reported any intimate partner violence compared with 43.1% for women in a current common-law relationship (*p *< 0.05) and 16.2% for women currently married (*p *< 0.001).

In Canada, unpartnered women who were victims of abuse were significantly more likely to use medications than women who were married (*p *< 0.01). No significant difference was found in medication use between unpartnered women and those in a current common-law relationship *(p *> 0.05).

Women with a common-law partner reported higher levels of lifetime sexual assault than unpartnered or married women. For example, Ontario respondents with a common-law partner reported lifetime sexual assault rates of 18.9%, compared with 11.0% among women currently living without a partner (*p *< 0.05) and 8.5% for women currently married (*p *< 0.01).

### Number of Children in the Household (Figure 13)

The rate of partner-perpetrated violence was not statistically different among respondents living in households with one child as compared with those with two or more children. These patterns were seen across all provinces and regions. Victims of violence living in households without young children were equally likely to report using medication as those with young children (versus one child, *p *> 0.05; versus two or more children, *p *> 0.05).

In Canada, the reported rate of lifetime sexual assault among respondents not currently living with young children was significantly lower than the rate among respondents with children (versus one child, *p *< 0.01; versus two or more children, *p *< 0.01).

### Education (Figure 14)

There were no consistent patterns seen in the analysis of respondents' educational level and the rate of partner violence. The rate of any partner violence (physical, sexual, emotional or financial) among all respondents was 23.0% for women with no education/some elementary or high school, 22.6% for women with high school diploma, and 19.6% for women with college or university education. Women with little schooling who were victims of violence were significantly more likely to use medications for anxiety, depression or sleep disturbance than women with high school (*p *< 0.05) or college or university education (*p *< 0.05).

Respondents with low educational levels were less likely to report lifetime sexual assault than those with higher levels of education. For example, in Canada overall, 8.9% of those with less than high school education reported lifetime sexual assault compared with 12.2% of women who were high school graduates (*p *< 0.01) and 13.0% for respondents with college or university education (*p *< 0.001).

### Household Income (Figure 15)

**Figure 14:**
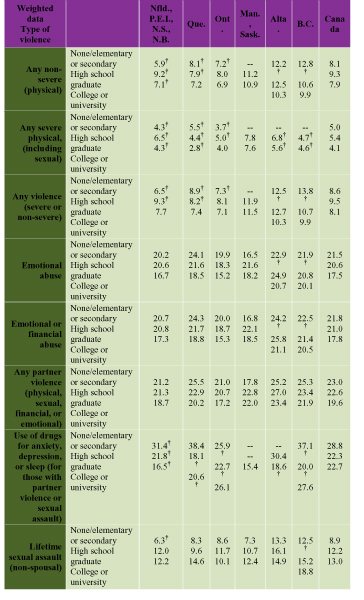
**Rate of Violence (%) Against Women by Partner or Ex-partner in the Previous 5 Years, by Educational Level of the Respondent (No Schooling, Some Elementary or Secondary, High School, College or University), Canada and the Regions, 1999. **Source: Statistics Canada, General Social Survey, 1999

Respondents living in low-income households were more likely to report all types of partner violence, and these patterns were seen in all provinces and regions (Figure 14). For example, in Ontario, 9.9% of respondents in households of less than $30,000 reported severe physical violence compared with 4.7% in households of $30,000–$49,999 (*p *< 0.05) and 2.8% in households of $50,000 or more (*p *< 0.01).

Women of all income groups reported emotional abuse, but rates of reported emotional abuse were highest for respondents in low-income households. In Alberta, among the lowest-income households 40.8% of respondents reported emotional or financial abuse, compared with 26.2% of households of $30,000–49,999 (*p *< 0.05) and 18.1% for households of $50,000 or more (*p *< 0.001).

Respondents from low-income households who reported partner violence were also more likely to use medication for anxiety, depression or sleep disturbance, patterns that were seen in all provinces and regions. In Canada, almost one in three women (31.4%) in households earning less than $30,000 reported medication use, compared with 20.3% of women in households of $30,000–49,999 (*p *< 0.01) and 18.9% for women in households of $50,000 or more (*p *< 0.001).

While levels of intimate partner violence differed across income groups, the rate of lifetime sexual assault was not statistically different for women across household income categories. For example, in Manitoba/Saskatchewan, the rate of lifetime sexual assault was 11.9% among women in households less than $30,000, 10.5% in households of $30,000–49,999 and 12.7% in households of $50,000 or more.

## Discussion

The GSS of 1999 has a number of limitations. First, most of the questions focus mainly on intimate partner violence. Second, most of the questions are based on a 12-month or 5-year window, not lifetime or childhood violence. Third, there are no questions on violence during pregnancy in the 1999 survey. Women in vulnerable situations who may have been abused, such as homeless women or those in institutions, are not included in the survey.

### Main Findings

In general, the rates of violence did not differ markedly across the provinces and regions. Rates were somewhat higher in Alberta and British Columbia and were somewhat lower in Ontario than those reported for all of Canada. However, the occurrence of violence (severe and non-severe) was high for all regions and provinces.

Nine per cent of Canadian women reported at least one violent episode by a current or previous partner in the previous five years. When weighted to the general population of Canadian women, this means that 673,000 Canadian women experienced at least one non-severe violent incident, 379,000 experienced severe violence, 1.5 million experienced emotional or financial abuse, and 1.4 million women had known sexual assault in their lifetime.

Severe violence was found less often than non-severe abuse. Nonetheless, about 4.8% of women reported being hit, kicked, beaten, choked, threatened with or having a knife or gun used against them, or forced into unwanted sexual activity, by a current or past partner. Lifetime sexual assault by someone other than a partner (which could include family members, non-family members or strangers) was reported by 11.6% of the women.

In the GSS survey, the youngest women reported experiencing the highest rate of abuse. Our Canadian data did not include enough subjects in the 15 to 19 year age group who had a partner. For young women 15 to 24 years of age, the rate of any violence by a partner was 42.4%. One possible explanation is that young women tend to date young men, a group with the highest rate of violence.

In the survey, those not born in Canada reported lower rates of intimate partner violence and lower rates of lifetime sexual assault. There were generally few differences in rates of intimate partner violence between visible minority women and non-visible minority women. Lifetime sexual assault, however, was less common among women who identified themselves as belonging to a visible minority. For many regions of the country, the number of respondents who classed themselves as member of a visible minority was small, and therefore the rates of violence could not be reported. Among those who did identify themselves as a visible minority or not born in Canada, the number of respondents who were from any particular country or region of the world was too small to undertake any sub-analyses for that group. It was also not possible to determine which respondents were refugees and which were immigrants. Immigrants may have different experiences of violence than refugees. Depending on the culture from which these women come, their experience of violence may be different from that of Canadian-born women. Some of these women may not consider some acts to be "violent." It is also possible that these women may be more reluctant to acknowledge or report violence.

Rates of violence were higher among Aboriginal than non-Aboriginal women. The small sample does not allow us to look at rates of violence among Aboriginal women in the eastern provinces, and since no data are provided for the northern territories, there was no information about women living in northern Canada.

No differences were found between women living in urban areas compared with their rural counterparts in the rate of intimate partner violence or lifetime sexual assault.

In the 1999 GSS, respondents were asked about "activity limitations," rather than disability *per se*. Disabled women are a known high-risk group for physical and sexual violence[62,63]. The findings of this analysis (for some of the provinces) support other studies showing that violence is greater among those with activity limitations. As well, these women had high rates of lifetime sexual assault.

In this study, among the highest rates of violence from a current or former partner were those experienced by women who were lone parents with children under 25 years of age. It was not possible to determine whether violence was a cause of the separation, but given the high rates of violence among lone mothers reporting violence from a former spouse, we can infer that violence may have been part of the reason for separation or divorce. This is supported by the findings that women who had a past partner reported the highest rates of intimate partner violence compared with women who lived with a common-law partner or were married.

The presence of children also seemed to be a factor in abusive households. The rate of violence was higher in households where there were young children than in those without young children present. It may be possible that the presence of children contributes to household stress, which, in turn, may contribute to intimate partner violence.

Women's education did not appear to be a factor in the experience of abuse, suggesting that women of all educational achievements can be victims of violence. In contrast, respondents living in low-income households were at higher risk of intimate partner violence but not lifetime sexual assault (which did not differ much across income groups).

## Conclusions

Although more research on violence against women is needed, there is a particular need for more information about the prevalence of violence among Aboriginal women, immigrant and refugee women from different populations, women with disabilities, and lesbian women, as well as about violence during pregnancy.

Violence against women (physical and sexual assault) is common. In the 1999 GSS survey, the individuals at highest risk were younger women (aged 15 to 24 years), Aboriginal women, those with activity limitations, lone parents with children under 25, those with a former partner and those living in a low-income household.

Violence against women is a health and health care issue. Women exposed to violence, whether as a child or an adult, are more likely to have physical health and mental health problems and to use more health care services. A history of violence contributes significantly to adverse health outcomes and to health care utilization. Experience of abuse escalates costs to the health care system.

### Policy Development and Data Requirements

• There is a need for further population-based surveys to identify high-risk groups and the prevalence of violence in these groups. It is crucial that population-based surveys include items that will enable us to determine the direct link between violence and health effects and the resource utilization of health care for these women. As well surveys about victimization or violence need to follow up with questions about health (not only injury).

• Further and extended research will be needed to identify a role for the health care system in the prevention, management and rehabilitation associated with violence. Future recommendations about programs or policies need to be based on valid, reliable and comprehensive empirical data.

• Screening for violence against women by health care providers has been suggested and is being carried out in some jurisdictions. However, there is little empirical evidence to support this approach; evaluation of violence screening programs is an identified research direction.

• Coordination across the country in terms of violence studies and violence programs would be very useful so that scarce resources are not used to duplicate efforts. Dissemination of successful programs (based on evidence) to other jurisdictions would aid in reducing duplicate efforts and allow resources to be used directly for program implementation and research.

• More research needs to be done in whether the identification of violence and treatment would result in lower health care utilization and improved health outcomes. Much more needs to be known about the health effects of violence, the costs to the health care system, and the role of the health care system in reducing violence.

## Figures and Tables

**Figure 6:**
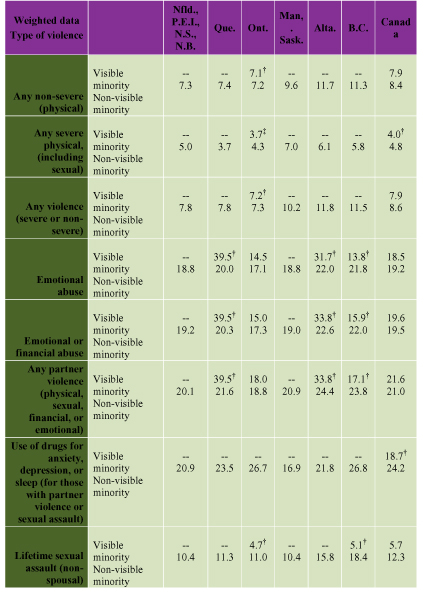
**Rate of Violence (%) Against Women by Partner or Ex-partner in the Previous 5 Years, by Self-identified Visible Minority Status, Canada and the Regions, 1999. **Source: Statistics Canada, General Social Survey, 1999

**Figure 8:**
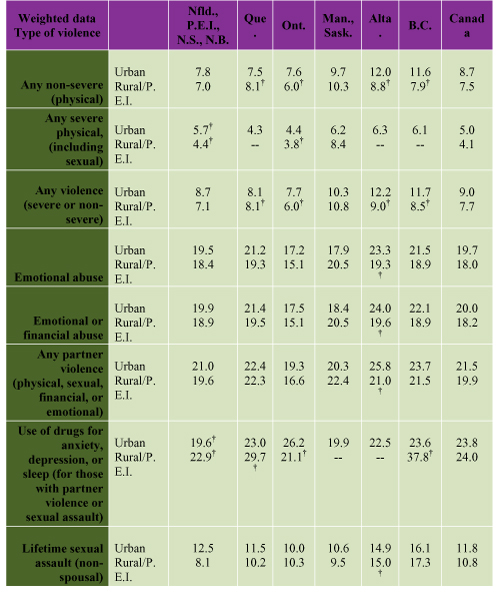
**Rate of Violence (%) Against Women by Partner or Ex-partner in the Previous 5 Years, by Urban/Rural Status, Canada and the Regions, 1999. **Source: Statistics Canada, General Social Survey, 1999

**Figure 12:**
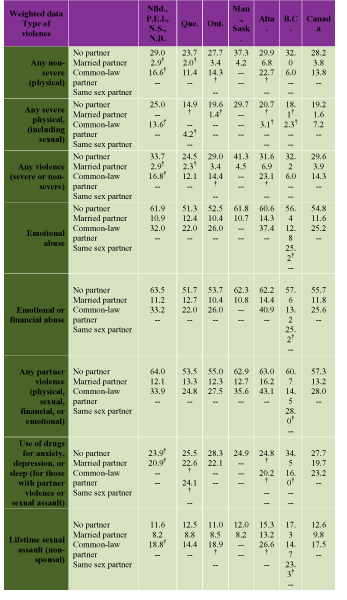
**Rate of Violence (%) Against Women by Partner or Ex-partner in the Previous 5 Years, by Presence or Absence of Partner in the Household, Canada and the Regions, 1999. **Source: Statistics Canada, General Social Survey, 1999

**Figure 13:**
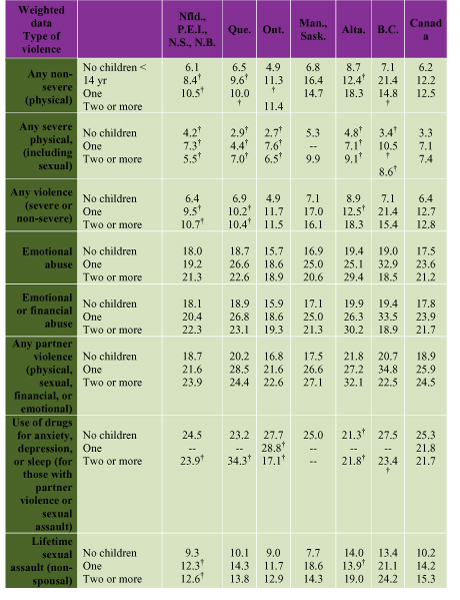
**Rate of Violence (%) Against Women by Partner or Ex-partner in the Previous 5 Years, by Number of Children Under 14 Years of Age Living in the Household, Canada and the Regions, 1999. **Source: Statistics Canada, General Social Survey, 1999

**Figure 15:**
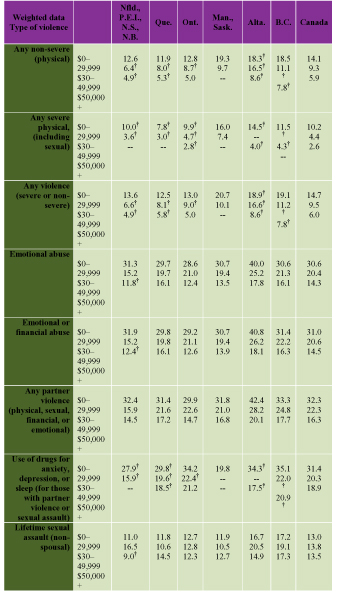
**Rate of Violence (%) Against Women by Partner or Ex-partner in the Previous 5 Years, by Household Income of the Respondents, Canada and the Regions, 1999. **Source: Statistics Canada, General Social Survey, 1999
